# Probing DNA clamps with single-molecule force spectroscopy

**DOI:** 10.1093/nar/gkt487

**Published:** 2013-06-19

**Authors:** Lin Wang, Xiaojun Xu, Ravindra Kumar, Buddhadev Maiti, C. Tony Liu, Ivaylo Ivanov, Tae-Hee Lee, Stephen J. Benkovic

**Affiliations:** ^1^Department of Chemistry, the Pennsylvania State University, University Park, PA 16802, USA and ^2^Department of Chemistry, Georgia State University, Atlanta, GA 30302, USA

## Abstract

Detailed mechanisms of DNA clamps in prokaryotic and eukaryotic systems were investigated by probing their mechanics with single-molecule force spectroscopy. Specifically, the mechanical forces required for the *Escherichia coli* and *Saccharomyces cerevisiae* clamp opening were measured at the single-molecule level by optical tweezers. Steered molecular dynamics simulations further examined the forces involved in DNA clamp opening from the perspective of the interface binding energies associated with the clamp opening processes. In combination with additional molecular dynamics simulations, we identified the contact networks between the clamp subunits that contribute significantly to the interface stability of the *S.cerevisiae* and *E. coli* clamps. These studies provide a vivid picture of the mechanics and energy landscape of clamp opening and reveal how the prokaryotic and eukaryotic clamps function through different mechanisms.

## INTRODUCTION

**Figure 1.**
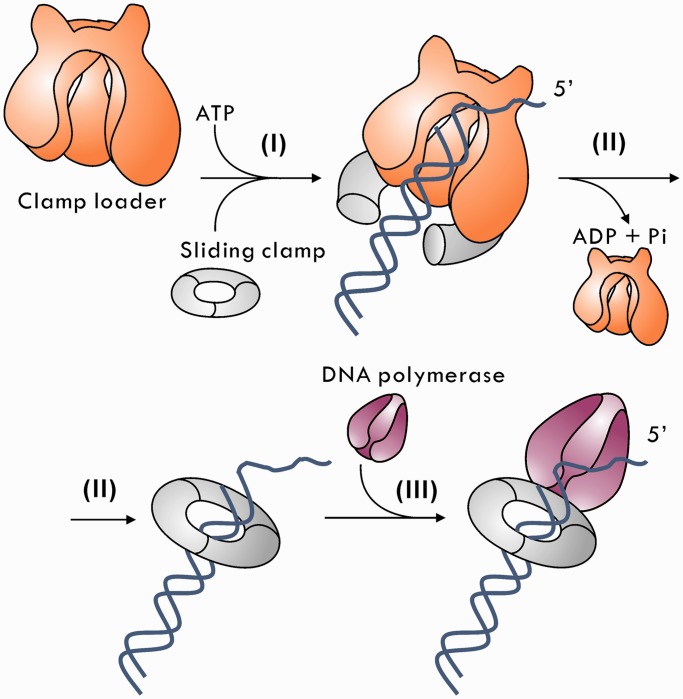
Clamp-loading mechanism and subsequent exchange of the clamp loader with DNA polymerase on the sliding clamp. In the presence of ATP, the clamp loader binds and opens the ring-shaped DNA sliding clamp for its loading on the DNA. Then the clamp loader hydrolyzes ATP to ADP before its dissociation from the clamp–DNA complex. In the final step, DNA polymerase binds the clamp and starts the DNA replication.

DNA sliding clamps play a pivotal role in DNA replication and repair (1–5), and, hence, cell reproduction and survival. Sliding clamps are linked to DNA with the assistance of a clamp loader (Figure 1). A DNA clamp also interacts directly with DNA polymerase, and thus facilitates the appropriate interaction between the polymerase and the DNA strand in processive DNA replication. Although the importance of DNA clamps has attracted great research interest, many fundamental mechanistic issues are still unresolved.

DNA clamps exist in all organisms and retain a well-conserved structure in all three domains of life (1). A crystal structure shows the yeast clamp loader RFC (replication factor C), a pentameric AAA+ adenosine triphosphatase (ATPase) (2–8), in complex with its corresponding clamp in a closed conformation (2). In that complex, RFC assumed a spiral arrangement with three of the five RFC subunits (RFC-A, RFC-B and RFC-C) in contact with the surface of the sliding clamp (PCNA; proliferating cell nuclear antigen). Furthermore, the interaction between the clamp loader and the clamp in solution is dependent on the presence of adenosine triphosphate (ATP). In the absence of ATP, RFC has a weak interaction (dissociation constant, *K*_d_ ∼20 nM) with PCNA. However, in the presence of ATP, RFC affinity with PCNA is increased by >10-fold (*K*_d_ ∼1 nM) (9). Recently, an archaeal complex examined by electron microscopy revealed an open clamp in complex with its clamp loader and DNA, suggesting an intermediate stage during clamp loading (4). The clamp loader assists in opening and subsequently closing the sliding clamp around DNA at primer-template junctions through an ATP-fuelled process. Therefore, the open clamp complex is believed to be an intermediate, where the PCNA ring is kept open before ATP hydrolysis by RFC that leads to subsequent ring closure. This is consistent with ensemble kinetic studies showing that sliding clamps can be opened by clamp loaders in the presence of either ATP or ATPγS, indicating that ATP hydrolysis is not required for clamp opening. Instead ATP hydrolysis is implicated in the clamp ring closure or clamp loader dissociation from the DNA (9,10).

In more detail, the yeast clamp loader binds two ATP molecules before binding the sliding clamp PCNA (11,12). Subsequently, the RFC/PCNA complex binds one more ATP molecule, followed by the binding of DNA and then an additional ATP molecule (13). Hydrolysis of ATP molecules (actual number of ATP molecules hydrolyzed per event is yet unclear) leads to the loading of the clamp onto DNA and the subsequent clamp loader dissociation from the clamp–DNA complex. The emerging model (10,14) involves the clamp loader binding three molecules of ATP to initiate a slow conformational change that enables the clamp loader to bind and facilitate the opening of the clamp. The clamp loader thus locks the clamp in an active loading conformation. ATP can effectively drive the clamp-loading process all the way to the formation of the closed yeast PCNA bound to DNA, whereas ATPγS cannot (15). Single-molecule data show that the process of yeast PCNA loading onto DNA involves passing through multiple conformational intermediates and is successful only after several failed attempts (16).

A recent theoretical study (17) showed that the clamp loader RFC selectively stabilizes the open conformation of the yeast PCNA over the closed. The complementary interface formed between RFC and the open ring PCNA compensates for the binding energy lost on PCNA subunit interface disruption. Furthermore, the barrier for the transition of the closed state to the open state was consistent with clamp opening occurring on a millisecond timescale. Although the equilibrium between the closed and open ring conformations of DNA clamps is known in favor of the closed conformation in solution, the actual force required to open the ring remains unresolved. To gain insights into the energies involved in DNA clamp opening and closing, we used single-molecule force spectroscopy and measured the mechanical force required for clamp ring opening using optical tweezers. We examined the *Saccharomyces cerevisiae* PCNA clamp, as well as the *Escherichia coli β*-clamp for comparison, as the clamp opening mechanism between yeast and *E. coli* has been suggested to be similar (1). However, we found that despite the structural and functional similarities, the eukaryotic clamp (*S. cerevisiae* PCNA) and its prokaryotic counterpart (*E. coli β*-clamp) make use of different ring opening mechanisms. Complementary theoretical studies further examined the interactions in the clamp subunit interfaces to determine the major contributors to the interface stabilities of DNA clamps.

## MATERIALS AND METHODS

### Construction of single-chain clamps

The *S. cerevisiae* PCNA’s native cysteines (C22/C30/C62/C81) were mutated to serines. A single-chain PCNA was cloned into the pET28a vector preceded by an N-terminal 6-His tag as described by McNally *et al.* (18). The C-terminal glutamate residues of the first and second PCNA subunits and the N-terminal methionine residues of the second and the third subunits in the chain were all deleted. The linker sequence between the first and the second subunits was GSNSQSNGSGA and the linker sequence between the second and the third subunits was GSNSQASNSGA. A cysteine was introduced by site-directed mutagenesis at K107 or D122 in the first subunit and F185 or D223 in the third subunits, respectively. Similarly, a single-chain *E. coli β*-clamp was cloned in the pET28 vector, and a cysteine was introduced at residue E64 or G102 in the first subunit and at residue E314 or E298 in the second subunit of the clamp. The linker sequence between the two subunits was GSNSQSNGSGA.

### Biotin and digoxygenin DNA handles

The *S. cerevisiae* PCNA gene was amplified from the pET22b PCNA plasmid using either 5′-Biotin-TTATTCTTCGTCATTAAATTTAGG-3′ or 5′-Dig-TTATTCTTCGTCATTAAATTTAGG-3′ and 5′-AmATGTTAGAAGCAAAATTTGAAG-3′. The 777-bp polymerase chain reaction (PCR) fragment was purified by a Qiagen PCR cleanup kit and quantified by NanoDrop™ spectroscopy. The 777-bp PCR fragment containing a biotin on one end and an amine group on the other end was reacted with a water-soluble amine-to-sulfhydryl linker (sulfo-SMCC, Thermo Scientific) at room temperature in buffer-A (20 mM sodium phosphate and 150 mM sodium chloride, pH 8.3). After 30 min of reaction time, the cross-linked DNA was purified quickly with a Qiagen PCR cleanup kit and then eluted with buffer-B (100 mM sodium phosphate and 150 mM sodium chloride, pH 7.2). The 777-bp PCR fragment containing a digoxigenin on one end and an amine group on the other was made and purified in a similar fashion. The DNA handles were prepared for immediate use.

### Coupling of DNA handles to clamps

We used sulfhydryl-maleimide chemistry to link maleimide-activated DNA linkers to PCNA cysteines. Single-chain PCNA was incubated at room temperature for 10 min in buffer B with a 100 M excess of Tris-(2-carboxethyl) phosphine (TCEP). Biotin and digoxygenin DNA handles were mixed in 1:1 ratio before being added to the TCEP-treated PCNA. The reaction mixture was incubated at room temperature for 30 min and then at 4°C overnight. The DNA handles were similarly attached to the *E. coli* single-chain *β*-clamp.

### Tethering of PCNA onto a quartz slide

The surface of a quartz slide was functionalized with biotin as reported previously (19). Then the slide surface was passivated with liposome followed by an extensive buffer wash. A solution of ∼50 nM streptavidin in buffer B was applied on the slide surface and incubated for 15 min before several rounds of washing with buffer B. A complex of PCNA or β-clamp with DNA handles was first incubated for 30 min with anti-digoxigenin–coated beads (2 µm) before being applied onto the functionalized quartz surface for incubation for >1 h to be tethered to the slide surface via biotin–avidin interactions.

### Force measurements by optical tweezers

A homebuilt optical tweezers set-up was used to measure the yeast PCNA rupture force (19). The quartz slide with the tethered PCNA was mounted on a custom-built computer-controlled x–y translation piezo stage. Anti-digoxigenin–coated beads were captured by the laser trap and tracked one at a time. The trap stiffness was 0.15 pN/nm, which was determined as published previously (19). Although the stage was moving at a designated speed (100 nm·s^−^^1^) to rupture the clamp interface, a video was recorded through a ×100 1.49 NA oil immersion objective by a charge-coupled device (CCD) camera. Traces of bead displacements from the center of the optical trap were extracted by processing the recorded videos. The displacements were then converted to the actual force applied by the optical tweezers on the clamps.

### Molecular dynamics simulation

The crystal structure of the *E. coli β*-clamp and yeast PCNA were obtained from the Protein Data Bank (PDB ID: 2pol and 1plq, respectively). Molecular dynamics (MD) simulations were performed using intact trimeric clamps. Subsequent molecular mechanics Poisson–Boltzmann surface area (MM-PBSA) calculations were carried out on reduced models generated from the aforementioned MD trajectories, which contained a single subunit interface. The truncated β-clamp model retained the N-terminal domain from the first subunit (residues 1–241) and the C-terminal domain (residues 122–366) of the second subunit. For the yeast clamp, we simply removed one of the equivalent subunits leaving a single interface between the other two remaining subunits. The xLeap module of AMBER 9 (20) was used to add hydrogen atoms to the models. All ionizable side chains were assigned to their protonation states at pH 7.0 using the WHATIF server (21). Each system was then solvated with TIP3P (22) water leaving a minimum distance of 10.0 Å from the protein surface to the edge of the simulation box. Counter ions were added to achieve charge neutralization, and additional 100 mM NaCl concentration was introduced to mimic physiological conditions.

The systems were then minimized for 5000 steps with fixed backbone atoms followed by 5000 steps of minimization with harmonic restraints (k = 15 kcal·mol^−^^1^·Å^−^^2^) to remove unfavorable contacts. All systems were then gradually heated to 300 K for 50 ps in the canonical ensemble (NVT) ensemble while keeping the protein backbone restrained. The equilibration was then continued for another 1.8 ns in the isothermal–isobaric ensemble (NPT) ensemble, and the harmonic restraints were gradually released in five stages.

Production runs were carried out in the isothermal isobaric ensemble (1 atm and 300 K) for 20 ns for all the systems. Long-range electrostatic interactions were evaluated with the smooth particle mesh Ewald (SPME) algorithm (23). For the short-range non-bonded interactions, we used a cut-off of 10 Å with a switching function at 8.5 Å. The integration time step was 2 fs, and the bonds between hydrogen and heavy atoms were fixed to eliminate the most rapid oscillatory motions in the system. The r-RESPA multiple time step method (24) was adopted with a 2-fs time step for bonded, 2 fs for short-range non-bonded interactions and 4 fs for long-range electrostatic interactions. All simulations were performed using the NAMD 2.7 code (25) with the AMBER Parm99SB parameter set (26) containing the force field for nucleic acids and proteins, on Hopper II, a Cray XE6 system at the National Energy Research Scientific Computing Center. Data were analyzed using the PTRAJ utility in AMBER (20) and custom VMD (27) TCL scripts.

### Steered molecular dynamics

For the steered molecular dynamics (SMD) simulation runs, we selected five conformations that are ∼2 ns apart along the MD production trajectories of the PCNA and β-clamp systems. These statistically independent, uncorrelated configurations were used to initiate constant velocity SMD simulations (28–33). External forces in SMD were applied on the following pairs of residues: K107-F185 and D122-D223 for yeast PCNA; E64-E314 and G102-E298 for the *E. coli β*-clamp. In each case, we selected the center of mass (COM) distance between the residue pair as our SMD pulling coordinate. The two residues were pulled away from each other by applying a harmonic restraint to their COMs (k = 25 kcal·mol^−^^1^·Å^−^^2^) and moving the restraint with constant velocity of 3 Å·ns^−^^1^ along the line connecting the COMs of the two residues. Configurations from the SMD pulling trajectories were sampled for analysis at an interval of 10 ps. To analyze the disruption of contacts at the subunit interfaces, we computed the number of hydrogen bonds as a function of pulling distance. For this hydrogen bond analysis, an H-bond distance cut-off of 3.2 Å and an angle cut-off of 30° were assumed. For each hydrogen bond detected during the pulling trajectory, the time of last occurrence was used to identify the contact break up. The interface opening mechanisms were then mapped onto the force-extension profile.

### Binding free energy calculation

The MM-PBSA method in AMBER 9.0 (20) was applied to calculate relative binding energies for the subunit interfaces of the β-clamp and yeast PCNA. The MM-PBSA calculations were carried out using the last 10 ns from the free MD trajectory for each clamp system. In total, 2500 frames were used for averaging. The free energy of binding can be calculated for each snapshot from the following expression:
(1)
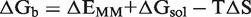

where ΔE_MM_ is the gas-phase molecular mechanic-binding energy, contributed by van der Waals and electrostatic interactions; ΔG_sol_ is the change in solvation free energy on subunit–subunit binding, comprising electrostatic and non-polar interactions; ΔS is the gas-phase entropy change on subunit–subunit binding. The electrostatic solvation energy is determined using the finite difference Poisson–Boltzmann (PB) method. In the PB calculation, a 0.5 Å grid size was used, and the dielectric constants of protein and water were set to 1.0 and 78.0, respectively. The non-polar contribution to the solvation free energy was determined from the solvent-accessible surface area according to the following equation:
(2)
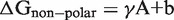

where A is the solvent-accessible surface area, and the solvation parameters γ and b are 0.0072 kcal·mol^−1^·Å^−2^ and 0 kcal·mol^−1^, respectively. The probe radius of the solvent was set to 1.4 Å. The surface area A was calculated using MolSurf in AMBER 9. The optimized set of atomic radii in AMBER 9 was used, and the atomic charges of the protein were taken from the ff99SB force field. It is important to note that the entropy contribution was not included; therefore, the computed binding energies should be considered only relative to one another.

The total binding energies were further decomposed into individual contributions from residues forming the subunit interfaces of yeast and *E. coli* clamps. The decomposition follows an established method to estimate the contributions of residues from each of the two subunits to the total binding energy by means of component analysis (34). The per residue binding energies are additive and include the same components as in Equation (1) except for the entropic contribution: van der Waals (ΔE_vdw_), electrostatic (ΔE_ele_) and solvation (ΔG_PBSA_) terms. The same dynamics trajectories used in the MM-PBSA calculations were used for energy decomposition. PTRAJ module of AMBER TOOLS 12 and VMD (27) was used for the analysis of trajectories and structural visualization.

## RESULTS AND DISCUSSION

### Construction of single-chain clamps

*Saccharomyces cerevisiae* PCNA is a homotrimer with three identical interfaces, whereas the *E. coli β*-clamp is a homodimer (1). However, both retain a ring-shaped structure with inner diameters sufficient to accommodate dsDNA. To measure the mechanical force required for clamp opening using optical tweezers, we constructed a single-chain clamp, which possesses only one subunit interface that can be opened, thereby allowing force measurement only on the targeted interface. Wild-type PCNA is composed of three monomers (A, B and C) aligned in a head-to-tail fashion (12), and a single-chain PCNA clamp was created by incorporating 10 amino acid linkers between the subunits A–B and B–C, leaving a single open interface between subunits A–C. In addition, we introduced a cysteine on subunits A and C by site-directed mutagenesis. These cysteines were used to attach DNA handles via sulfhydryl-maleimide chemistry. Previously, McNally *et al.* (18) have reported that the single-chain PCNA generated by this method is structurally identical to wild-type PCNA while retraining full activity. We used the enhanced ATPase activity of RFC in the presence of the clamp as an assay for clamp functionality (9). We measured the ATPase activity of the clamp loader in the presence of the single-chain clamp using an enzyme-coupled reaction in which the production of Adenosine diphosphate (ADP) was coupled to the depletion of nicotinamide adenine dinucleotide (reduced form) (NADH) by pyruvate kinase and lactate dehydrogenase (35). We found that the ATPase activity of the clamp loader was stimulated by the single-chain clamp to the same extent as the wild-type clamp. Similarly, we constructed a single-chain *E. coli β*-clamp that exhibited the same activity as the wild-type β-clamp as measured by the ATPase assay. Results from these ATPase assays together with previous research (12,16,18) indicate that the modification to the residue sites is unlikely to perturb the clamp stabilities.

### Rupture force measurement

**Figure 2.**
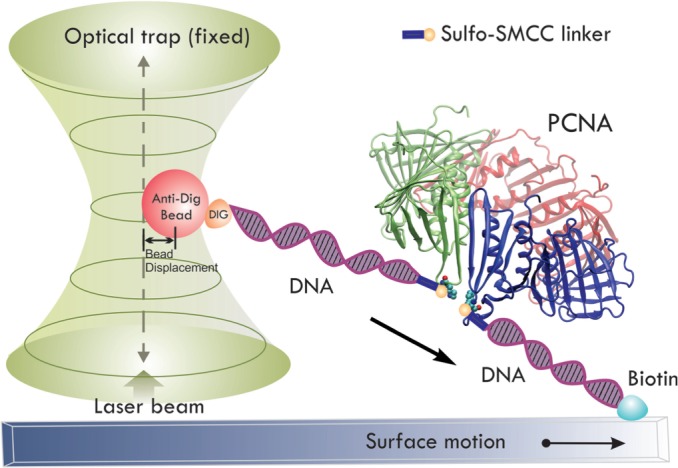
Schematic representation of clamp rupture force measurement with optical tweezers. One end of a single-chain PCNA linked to a DNA linker is tethered to the quartz slide via a biotin–avidin interaction and on the other end an anti-dig bead is attached to a dig-DNA linker. The bead is trapped at the center of a fixed laser beam. The PCNA is pulled by moving the microscope stage at a constant speed (100 nm·s^−1^). Displacements of the bead from the optical trap center were recorded by an EMCCD camera. An abrupt decrease in the bead displacement from the trap center is observed on a rupture event.

**Figure 3.**
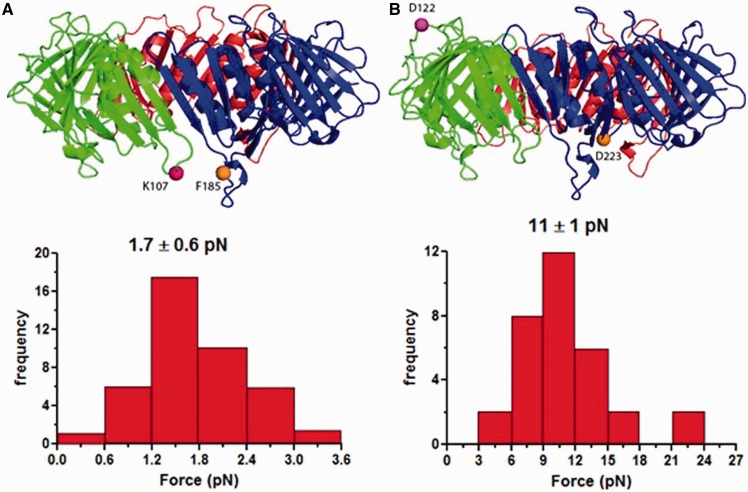
Histograms of PCNA interface rupture force. Multiple PCNA pulling experiments were performed at different sites; the rupture forces and their corresponding frequencies were plotted in the histogram. (**A**) PCNA pulled at residues K107 and F185. The interface was ruptured at 1.7 ± 0.6 pN. (**B**) PCNA pulled at residues D122 and D223. The interface was ruptured at 11 ± 1 pN.

We measured the mechanical force required for the PCNA opening using optical tweezers to gain a better understanding of the forces and energy involved in DNA clamp opening and closing. One end of the single-chain PCNA was attached to a DNA linker tethered to a quartz slide surface via a biotin–avidin interaction, whereas the other end was attached to a DNA linker capped with an anti-digoxigenin–coated bead (Figure 2). Typical traces manifesting rupture events are depicted in Supplementary Figure S1. The abrupt decreases in the force or displacement of the bead from the trap center indicate the rupture events of clamp opening. Multiple traces for the rupture events were collected, and the force histograms were constructed (Figure 3A and B) for determining the average bead displacements. The rupture force involved in PCNA ring opening was calculated by multiplying the averaged bead displacements by the trap stiffness, 0.15 pN·nm^−^^1^. When PCNA was pulled at residues K107 and F185, we observed that 1.7 ± 0.6 pN was sufficient for the PCNA ring opening. In contrast, when pulling PCNA at residues D122 and D223, a substantially higher rupture force, 11 ± 1 pN, was required. A control experiment using only a 1-kb DNA linker with an anti-dig bead on one end and biotin on the other was performed to confirm that the observed rupture forces are due to the clamps (Supplementary Figure S2). In these control experiments, no rupture events were observed within the operative regime of the optical trap.

**Figure 4.**
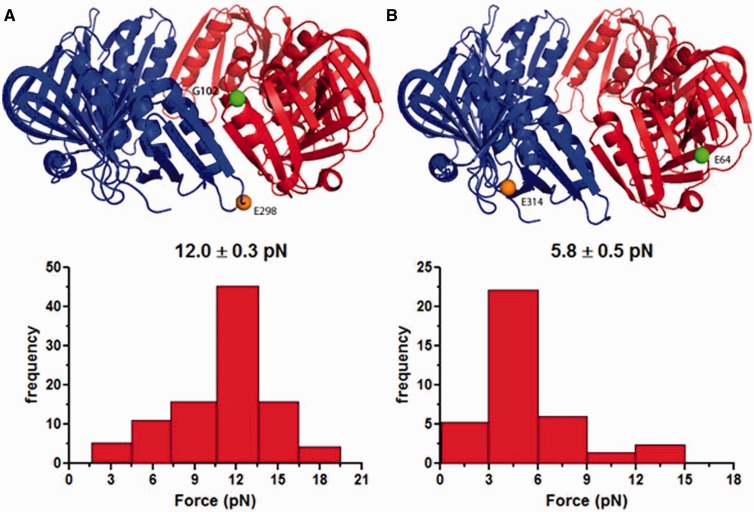
Histograms of the *β*-clamp interface rupture force. Multiple *β*-clamp pulling experiments were performed at different sites; the rupture forces and their corresponding frequencies were plotted in the histogram. (**A**) *β*-clamp pulled at residues G102 and E298.The interface was ruptured at 12.0 ± 0.3 pN. (**B**) *β*-clamp pulled at residues E64 and E314. The interface was ruptured at 5.8 ± 0.5 pN.

The force experiments on the *E. coli β*-clamp were performed in the same manner, and histograms of the data are presented in Figure 4A and 4B. When the β-clamp was pulled at residues E64 and E314, 5.8 ± 0.5 pN was sufficient for ring opening, whereas pulling at residues G102 and E298 required a higher rupture force of 12.0 ± 0.3 pN. Comparison of the minimum force required for yeast PCNA opening versus *E. coli β*-clamp opening shows that the *E. coli β*-clamp requires at least three times larger opening force as compared with yeast PCNA.

The data show that in both systems, the mechanical force required for clamp opening was dependent on the position of attachment. This can be explained by inspecting the locations of the attachments. Smaller rupture forces were observed when the pulling force was applied directly near the subunit–subunit interface (PCNA: K107-F185, Figure 3A; β-clamp: E64-E314, Figure 4A). Larger rupture force was required when the pulling force was applied away from the subunit–subunit interfaces (Figure 3B and 4B), which might be due to disruptions of other interactions outside of the subunit interface. Thus, the smaller force values are more accurate assessments of the forces necessary to disrupt the interactions in the clamp subunit interfaces.

### Interface interaction energies

To understand differences in the force required to disrupt the subunit interface between *S. cerevisiae* PCNA and *E. coli β*-clamp, we examined the various components of the interaction energy of the subunit–subunit complexes using MM-PBSA analysis (Supplementary Table S1). Unlike the case of MM-PBSA applied to small ligands, it is difficult to accurately assess the entropic contribution to binding. Calculating the entropic component from normal mode analysis would typically lead to large standard deviations for the binding free energies. Therefore, we opted to focus solely on the enthalpic contribution to provide quantitative comparison of the subunit–subunit interfaces between the PCNA and β-clamp. Thus, the values in Supplementary Table S1 represent binding energies rather than binding free energies. As expected, because of lack of entropy–enthalpy compensation, the absolute binding energies are overestimated compared with the corresponding experimental free energies. Nonetheless, previous applications of MM-PBSA to protein interfaces demonstrated the usefulness of comparing the relative binding enthalpies and identifying binding hotspots based on energy decomposition (36).

The MM-PBSA analysis for our complexes leads to substantial negative-binding energies for the clamp interfaces in both the *E. coli β*-clamp and *S. cerevisiae* PCNA. This is consistent with the experimental evidence suggesting that both clamps remain closed in solution (37), and that the closed ring conformation of the clamp is more thermodynamically favorable than the open conformation. Furthermore, the binding energy ΔG_b_ for the *E. coli β*-clamp interface was found to be four times larger than the corresponding energy for the yeast PCNA interface (Supplementary Table S1). The difference is significant (even when we take into account the neglect of the entropic effects). This is in agreement with the optical trap rupture force measurements, which found that the subunit–subunit interface of β-clamp requires a greater force to disrupt than that of yeast PCNA.

More importantly, the factors contributing to subunit–subunit interface stability were found to be entirely different between the β-clamp and PCNA, which might explain the difference in the rupture force observed in the optical trap experiments. First, the individual components of the binding energies were compared with each other (Supplementary Table S1). The intermolecular (gas-phase) van der Waals interactions are favorable and contribute almost equally to the stability of both interfaces. However, the gas-phase electrostatic interactions are favorable for the *E. coli β*-clamp interface and unfavorable for the yeast PCNA interface. The non-polar solvation energy (ΔG_non__-__polar_) corresponds to the burial of solvent-accessible surface area (SASA) and contributes favorably to the stability of both clamp interfaces. Another dissimilarity is the electrostatic solvation energy (ΔG_polar_), which disfavors the formation/association of the *E. coli β*-clamp interface, whereas the ΔG_polar_ term greatly favors the formation of the yeast PCNA interfacial interactions. Thus, there seems to be a compensation effect between the gas-phase electrostatics and the electrostatic solvation energy, where these two electrostatic terms act in opposite directions for each clamp interface. The gas-phase electrostatics are the dominant contributor to the formation of subunit interface in both cases, where the binding in the yeast clamp is disfavored, whereas the binding in the β-clamp is favored. This outcome can be rationalized by the fact that the opposing sides of the yeast PCNA subunit interface are both negatively charged, whereas the β-clamp interface is composed of an anti-parallel β-sheet with positive and negative residues sequestered on each strand, forming salt bridges across the interface. Such salt bridge interactions, found in the context of largely hydrophobic environment, contribute to far greater overall stability of the β-clamp when compared with the PCNA clamp. Even though the MM/PBSA overestimates the absolute value of binding free energy because of the omission of entropy in the calculation, the trends observed from simulations are consistent with the optical trap force experiments.

**Figure 5.**
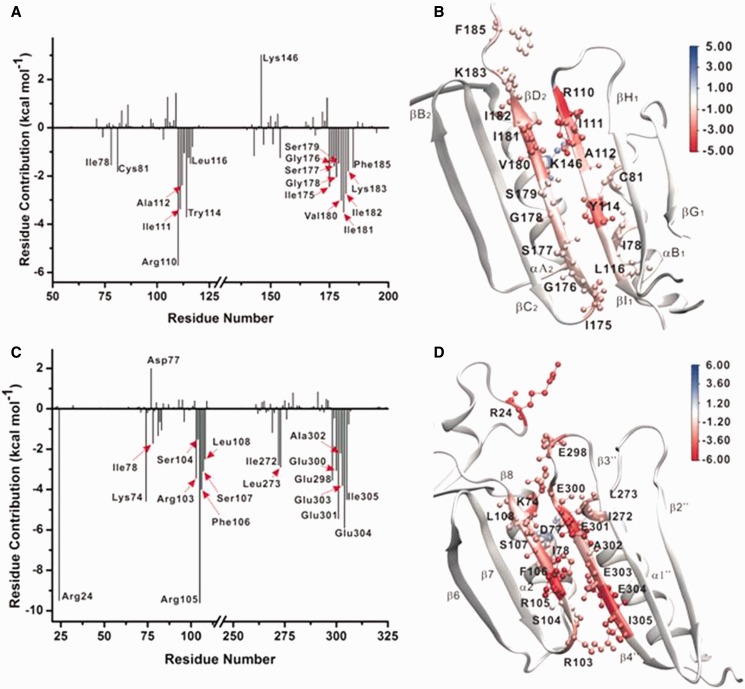
Binding energy contributions from the interface residues in (**A** and **B**) yeast PCNA and (**C** and **D**) *E. coli β*-clamp. Individual residue contributions are indicated on the graphs, and residues contributing more than ± 1.5 kcal·mol^−1^ are labeled explicitly. The binding energy contributions are also mapped onto the structures of the two interfaces and are colored from red (negative) to blue (positive).

The total binding energy at each subunit–subunit interface was further decomposed into contributions from individual residue as shown in Figure 4. Residues contributing more than ±1.5 kcal/mol toward the stability of each interface are labeled explicitly (Figure 5A and C). The residues contributions are also mapped onto the structure of the two subunit interfaces and color-coded from positive (blue) to negative (red) in panels 5B and 5D.

For the PCNA clamp, the dominant contribution to the binding enthalpies comes from residues positioned along the interfacial antiparallel β-strands (Ile175, Gly176, Ser177, Gly178, Ser179, Val180, Ile181, Ile182, Lys183 and Phe185 on strand βD2, and Ile78, Cys81 Arg110, Ile111, Ala112, Tyr114 and Leu116 on βI1). The subunit interface is largely hydrophobic, and, in this hydrophobic context, stability is primarily determined by the presence of seven main chain hydrogen bonds. Notably, for the yeast PCNA, the residues with greatest contributions to stability are not evenly distributed along the length of the β strands, giving rise to the possibility of asymmetric disruption of the interface on opening. In such a scenario, the interface would preferentially unzip from one end of the β-sheet, where the residue contacts are less stable.

The α-helices at the back of the anti-parallel β-sheet, facing the central hole of PCNA (αB1 and αΑ2), were found to contribute to a far lesser extent to the subunit–subunit interface stability. Favorable contributions arise from residues Ile78, Cys81 on helix αB1 and to a lesser extent Leu151 and Leu154 on the opposing αΑ2 helix. Furthermore, Lys146 on αΑ2 helix was found to destabilize the interface by 3.02 kcal·mol^−^^1^ because of unfavorable electrostatic interaction with the Arg110 on the opposing βI1 strand.

The β-clamp interface is organized differently from the PCNA interface with electrostatic interactions playing a much more prominent role in the β-clamp interface. Five glutamate residues, Glu298, Glu300, Glu301, Glu303, and Glu304, are all positioned along the interfacial β-strand β4′′. Arg103 and Arg105 from the β8 strand and Lys74 from the α2 helix are all segregated across the interface from the negative residues. This residue placement/pattern contributes greatly to the total subunit–subunit binding energy (Figure 4B). Meanwhile, hydrophobic residues such as Ile78 on α2, Leu108 on β8, Ile272 and Leu273 from α1′′ significantly stabilize the interface through hydrophobic interactions. Residues Phe106, Ser107 and Leu108 on β8, Ala302 and Ile305 on β4′′ also contribute to interface stability by forming a hydrophobic core and through backbone hydrogen bonds across the β8–β4′′ sheet. Additionally, Arg24 on a distant loop connecting α1 and β2 seems to stabilize the interface, most likely through electrostatic interaction with the negatively charged Glu298 on the β4′′ strand.

### Interface rupture mechanisms

Sliding clamp opening is an activated process. Activated rare events are not commonly accessible through straightforward MD simulations. To enforce separation of the two interfacial β strands of PCNA and β-clamp, we relied on SMD: a method that involves the use of a harmonic constraint moving at a constant velocity to ‘steer away’ the centers of mass of groups of atoms, and thus promote enhanced sampling. SMD was used to force open the clamp subunit–subunit interfaces by applying external forces to steer the centers of mass of the selected residue pairs along the direction connecting the residue COMs. A force constant k of 25 kcal·mol^−^^1^·Å^−^^2^ was sufficient to open the interfaces of either the PCNA or β-clamp. The steering velocity v was selected to be 3.0 Å·ns^−^^1^. SMD simulations probed the energy barriers for interface disruption in a way that is analogous to single-molecule optical tweezers experiments. However, the time scales involved in computational pulling runs are much shorter than the corresponding experimental timescales. As a result, the computed force-extension profiles include a sizable contribution from non-equilibrium effects, which is not present in the corresponding experimental profiles. Therefore, although the rupture forces determined by the SMD simulations are not numerically comparable with the experimental values, the mechanistic details regarding subunit interface disruption under external forces can be analyzed, and the relative magnitude of the forces between different systems can be compared. Residue contacts along the interface break in stages leading to a characteristic seesaw pattern (several peaks and shoulders). Integrating the force/extension plot yields work versus distance profile.

In all five runs, PCNA pulled at K107 and F185 completely opened up after ∼40-ns simulation. For β-clamp pulled at G102 and E298, only one of five runs resulted in the complete clamp opening after 50 ns. Although the other four β-clamp systems eventually open up, these systems exhibited contact breaking and reformation between residues not involved in the original interface. Force-extension profiles from the run that opened the β-clamp without complications and from the run that yielded the lowest work profile for the PCNA clamp are illustrated in Figure 5. The force applied by the virtual spring on the pulling residue accrues on the extension until a threshold is reached, as indicated by the peaks in the plots, which breaks the interactions between the subunits and results in subsequent force decrease. The difference in the stability of the yeast and *E. coli* clamp interfaces is evident from the fact that the yeast clamp requires a smaller maximum force to open when compared with the β-clamp. The major peak on the β-clamp profile is 15.52 kcal·mol^−^^1^·Å^−^^1^, ∼3.3 times larger than the corresponding peak of the yeast clamp profile (4.74 kcal·mol^−^^1^·Å^−^^1^). This finding is in good correspondence with the outcome of the MM-PBSA analysis and the experimental force data (Figure 3 and 4).

**Figure 6.**
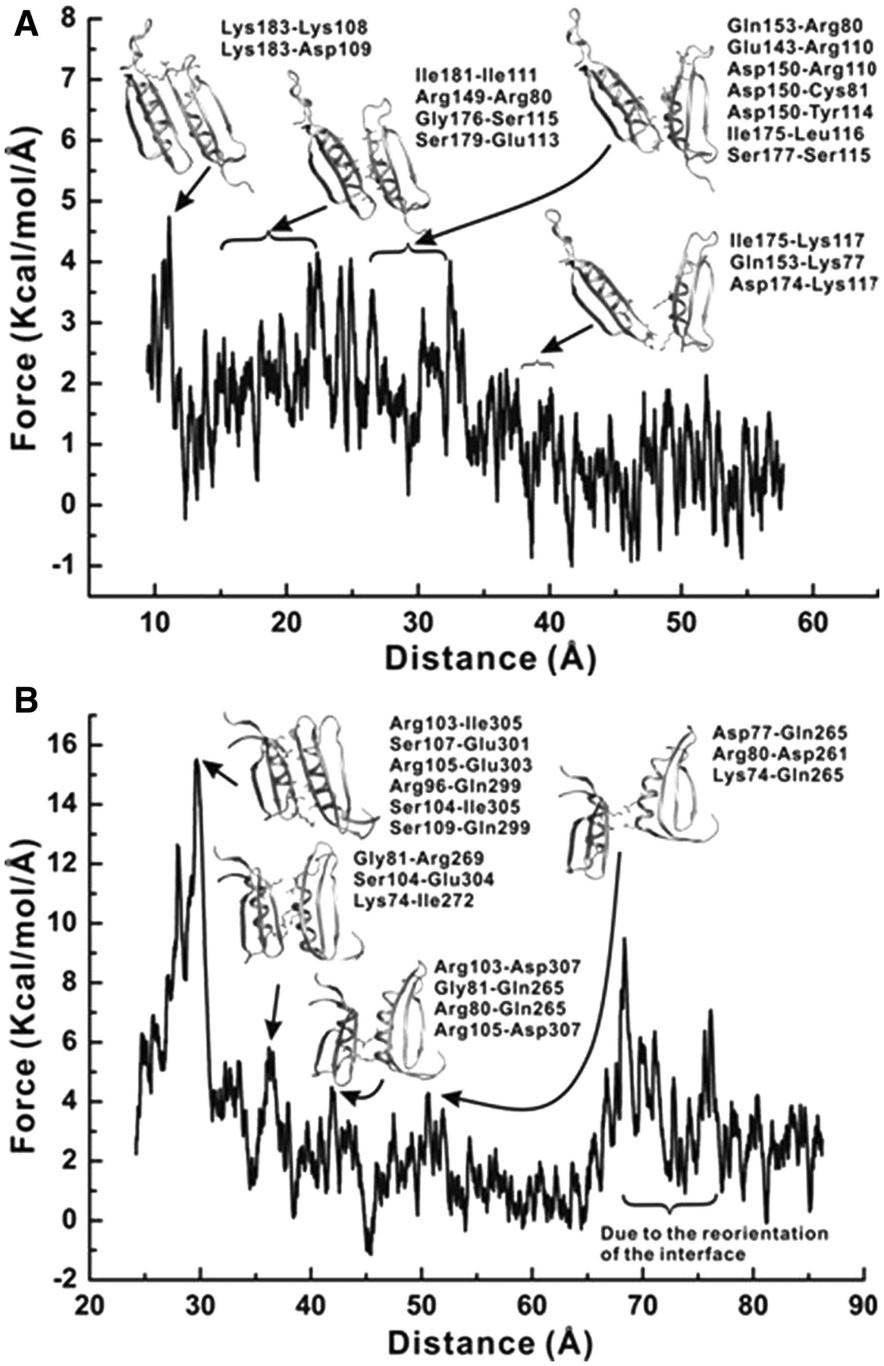
Interface disruption mechanism as determined from the SMD force-extension profiles for (**A**) yeast PCNA and (**B**) *E. coli β*-clamp. The force-extension profile is smoothed by adjacent averaging for a 100-frame window. Representative snapshots of the subunit interfaces at different stages of separation are shown. Arrows indicate the correspondence to distinct peaks in the force-extension profiles.

The structural differences at the subunit interfaces in yeast PCNA and *E. coli β*-clamp lead to different opening mechanisms in the SMD trajectories. Four distinct groups of residue contacts get disrupted consecutively along the yeast PCNA pulling trajectory, with corresponding peaks readily identified in the force extension profile (Figure 6A). Specifically, yeast PCNA was found to open by gradual unzipping of the interfacial anti-parallel β-sheet βD2–βI1. The strand separation is initiated from the end of the interface holding the residue pair K107-F185. Interestingly, this end is presumed to be more stable based on the MM-PBSA-binding energy decomposition analysis. This suggests that the exact position where the external forces are applied matters for the observed directionality of opening. This finding is also consistent with the results from the optical tweezers experiments, which showed that the maximum rupture force is dependent on the positions of the residue pair being pulled.

Unlike the yeast PCNA, the β-clamp interface is stabilized primarily by residues in the middle of the antiparallel β-sheet β8–β4′′. Its strength is derived from the presence of a hydrophobic core as well as main chain hydrogen bonds and salt bridge interactions. The SMD pulling run reveals a much more abrupt opening, whereby a large number of contacts are broken cooperatively (as indicated by a prominent initial peak in the force-extension profile in Figure 6B). The remaining few contacts in the middle of the interface correspond mainly to charged contacts between positive and negative residues on the opposing sides of the interface. These contacts are the last to be disrupted simply because of the long side chains of Arg residues that allow substantial extension before breaking contact.

### Energy requirements for clamp opening

SMD simulations afforded the pulling distances until the clamps were fully opened (Figure 6). For the yeast PCNA, the clamp-opening distance was estimated to be 35 Å, and for the *E. coli β*-clamp it was 31 Å. This is consistent with the experimental data for the yeast PCNA clamp-opening distance (34 Å) determined from a previous fluorescence resonance energy transfer (FRET) study (15,38). By combining the clamp-opening distances derived from the SMD simulations and the rupture forces measured by optical tweezers, we can approximate the work necessary for rupturing the subunit interfacial interactions. From our data, we estimated the maximum work spent on rupturing the bonds with minimum-binding energies. The rupture forces measured by force spectroscopy were 1.7 ± 0.6 pN at residues K107 and F185 for yeast PCNA and 5.8 ± 0.5 pN at residues E64 and E314 for *E. coli β*-clamp. The rupture work can be determined by multiplying the rupture force by the distance change at the interface on rupture. Thus, the work required for clamp-opening is ∼0.86 kcal·mol^−^^1^ for yeast PCNA and 2.58 kcal·mol^−^^1^ for *E. coli β*-clamp. It should be noted that the clamp-binding energies must be smaller than the calculated values aforementioned, as the magnitude of any experimentally measured rupture work should always be larger than the equilibrium-binding energies.

The overall clamp loading process is driven by ATP hydrolysis; therefore, naturally it is not unreasonable to expect that most of the energy released by ATP hydrolysis should be used for clamp opening. However, based on the number of hydrolyzed ATPs estimated from ensemble kinetic studies (10,14), there exist huge disparities between the energy generated by ATP hydrolysis and the energy required to open the clamp. In other words, only a small fraction of the energy from ATP hydrolysis is needed to open the clamps. This is consistent with literature data, suggesting that ATP is more important in activating the clamp loader to bind and stabilize the otherwise short-lived high energy open clamp conformation (37,38). Thus, it is possible that the actual force (or the energy) required to directly disrupt the subunit–subunit clamp interface is relatively small.

The kinetic advantage of the complex for clamp loading is well illustrated by previous single-molecule kinetic studies by FRET (16), in which several attempts are required before the clamps are fully loaded on DNA. During clamp loading, a long-lived clamp loader-clamp complex will have more opportunity to survive failed attempts and, hence, enhance the overall efficiency of clamp loading onto the DNA strand. Consequently, ATP hydrolysis may be ultimately associated with dissociation of the clamp loader in addition to closure of the clamp (39,40).

## CONCLUSIONS

Here, we probed the mechanics of clamp loading from the perspective of the forces and energy required for opening the subunit interfaces for both *S. cerevisiae* PCNA and *E. coli β*-clamp. In addition, we analyzed specific hydrophobic and polar contacts from MM-PBSA analysis, as well as delineated the detailed mechanism of disruption of the interfaces in the two clamp systems. We found distinct opening mechanisms—gradual unzipping in the case of yeast PCNA versus abrupt cooperative disruption for *E. coli β*-clamp. These differences are dictated by the nature of the contacts formed at the subunit interfaces. In comparison with yeast PCNA, *E. coli β*-clamp interface exhibited a far greater number of strong hydrophobic interactions in addition to favorable electrostatics and strong specific salt bridge interactions. These differences in the dominant interactions result in a corresponding large overall difference in interface stabilities, and thus in the mechanical forces required for clamp opening between the yeast and the *E. coli* clamps.

Furthermore, we found that little energy is required to open both clamps, whereas *E. coli β*-clamp seems to require at least three times more force and energy to open than yeast PCNA. This is consistent with the suggestion that yeast PCNA can transiently open on a millisecond time scale, and the ATP-activated clamp loader functions to stabilize the high-energy open form of the clamp by trapping it in a complex (10). On the other hand, although the closed *E. coli β*-clamp is more stable than the closed yeast PCNA as determined by the dissociation lifetimes of the rings on circular DNA molecules (16), there is evidence indicating that the *E. coli* clamp loader γ complex might actively participate in the opening of β-clamp (39). Both the higher stability of the closed ring conformation and possible need for an active clamp loader participation in the ring opening are supported by the higher force and energy cost found here for disruption of the subunit interfacial interactions in β-clamp. Thus, despite the fact that *S. cerevisiae* PCNA clamp and *E. coli β*-clamp share many similar mechanistic aspects (40) in the clamp loading processes, there are many important distinctions between the two systems, such as the force and its associated detailed mechanisms of ring opening, which would elude the more common ensemble-averaged mechanistic studies.

## SUPPLEMENTARY DATA

Supplementary Data are available at NAR Online: Supplementary Table 1 and Supplementary Figures 1 and 2.

## FUNDING

Funding for open access charge: National Institute of Health [NIH GM013306 to S.J.B.] National Science Foundation [NSF-CAREER MCB-1149521 to I.I.], start-up funds from Georgia State University (to I.I.). Computational resources were provided in part by allocations from the NSF XSEDE program [CHE110042]; National Energy Research Scientific Computing Center supported by the DOE Office of Science [contract DE-AC02-05CH11231].

*Conflict of interest statement.* None declared.

## Supplementary Material

Supplementary Data
