# Supplementary material for: Probing DNA clamps with single-molecule force spectroscopy
Source: Nucleic Acids Res. 2013 Jun 19;41(16):7804–14. doi: 10.1093/nar/gkt487 (PMC3763527; doi:10.1093/nar/gkt487)
Supplement: Supplementary Data [file supp_41_16_7804__index.html]

Probing DNA clamps with single-molecule force spectroscopy — Probing DNA clamps with single-molecule force spectroscopy — Supplementary Data 

# Probing DNA clamps with single-molecule force spectroscopy

## Supplementary Data

files

**Files in this Data Supplement:**

- Supplementary Data - doc file
