# Supplementary material for: Probing DNA clamps with single-molecule force spectroscopy
Source: Nucleic Acids Res. 2013 Jun 19;41(16):7804–14. doi: 10.1093/nar/gkt487 (PMC3763527; doi:10.1093/nar/gkt487)
Supplement: Supplementary Data [file supp_gkt487_nar-00753-m-2013-File008.doc]

Supplementary Data


Probing DNA clamps with single-molecule force spectroscopy

Lin Wang1, Xiaojun Xu2, Ravindra Kumar1, Buddhadev Maiti2, C. Tony Liu1, Ivaylo Ivanov2,*, Tae-Hee Lee1,* and Stephen J. Benkovic1,*


1	Department, Institution, Town, State, Postcode, Country Department of Chemistry, the Pennsylvania State University, University Park, PA 16802

2	Department of Chemistry, Georgia State University, Atlanta, GA 30302

* To whom correspondence should be addressed. Stephen J. Benkovic, Tel: +1 (814) 865-2882; Fax: +1 (814) 865- 2973; E-mail: sjb1@psu.edu; Ivaylo Ivanov, Tel: +1(404) 413-5529; E-mail: iivanov@gsu.edu; Tae-Hee Lee, Tel.: +1 (814) 867-2232; E-mail: txl18@psu.edu

Table S1. Free Energy Analysis (kcal mol-1) for the formation of clamp subunit interfaces
Contribution	PCNA	â-clamp	
ÄEele	2138.80 ± 58.24	-486.51  ± 36.39	
ÄEvdw	-73.95 ± 4.37	-60.99 ± 5.93	
ÄGnonpolar	-11.28 ± 0.45	-11.19 ± 0.52	
ÄGpolar (PB)	-2076.70 ± 59.39	463.11 ± 32.38	
ÄGsol (PB)a	-2087.98 ± 59.22 	451.92 ± 32.17	
ÄGele (PB)b	62.09 ± 8.32	-23.40 ±10.45	
ÄÄGb (PB)	-23.14 ± 7.64	-95.58 ± 8.25	
aThe polar/nonpolar (ÄGsol = ÄGpolar + ÄGnonpolar) contribution. bThe electrostatic (ÄGele = ÄEele + ÄGpolar) contributions.


Figure S1. Ten force-extension curve examples. The DNA-clamp complex was pulled at a constant speed (100 nm/s). The applied force was calculated by multiplying the bead displacement by the trap stiffness (0.15 pN·nm-1). The extension is calculated by subtracting the bead displacement from the stage displacement and post-synchronized to be zero at the moment of rupture.


Figure S2. Typical force extension curves of the 1kb DNA linker with an anti-dig bead on one end and biotin on the other. No rupture events were observed within the operative regime of the optical trap (0 - 40 pN).
